# MR1‐ and MAIT cell‐dependent pathology in a mouse model of AD

**DOI:** 10.1002/alz.087711

**Published:** 2025-01-03

**Authors:** Randy R Brutkiewicz

**Affiliations:** ^1^ Stark Neurosciences Research Institute, Indiana University School of Medicine, Indianapolis, IN USA

## Abstract

**Background:**

We have found that the MHC class I‐like MR1 molecule and the innate‐like T cells that recognize them (MAIT—mucosal‐associated invariant T cells) contribute to the development of pathology in Alzheimer’s disease (AD). Moreover, we find elevated levels of MR1 expression and increased numbers of MAIT cells in the brains of 5XFAD mice overtime; these MAIT cells express high surface levels of the T cell activation markers, CD25 and CD69. Here, we are focused on how these two phenomena are connected in AD pathology.

**Method:**

We will analyze mice (MAIT^CAST^) that have up to 20X the normal level of MAIT cells crossed onto the 5XFAD background and/or those expressing transgenes that encode GFP or YFP under the control of various cytokine promoters. Flow cytometry will allow us to determine the fraction of MAIT cells producing proinflammatory cytokines and their numbers, as well as their level of T cell activation markers as compared to conventional T cells, in both brain and peripheral immune organs.

**Result:**

In normal 5XFAD mice, we found that, not only are MAIT cell higher in the brain, but also in the liver. Moreover, we have found that MAIT^CAST^ mice have up to 20X the normal level of MAIT cells in the brain, as well in peripheral tissue, such as the liver.

**Conclusion:**

Our studies have identified an important role for the MR1/MAIT cell axis in AD pathology, and its potential utility as a novel therapeutic target for AD patients.

